# Smokers present a three times higher risk of graft failure after ACL reconstruction: A single‐centre retrospective analysis

**DOI:** 10.1002/jeo2.70422

**Published:** 2025-09-10

**Authors:** Filip R. Hendrikx, Pietro Conte, Annemieke Van Haver, Kristien Vuylsteke, Koen Carl Lagae, Peter Verdonk

**Affiliations:** ^1^ ORTHOCA Antwerp Belgium; ^2^ Department of Orthopaedic Surgery Antwerp University Hospital (UZA) Antwerp Belgium; ^3^ IRCCS Humanitas Research Hospital Milan Italy; ^4^ Department of Biomedical Sciences Humanitas University Milan Italy; ^5^ MoRE Institute Antwerp Belgium; ^6^ ASTARC Department Antwerp University Antwerp Belgium; ^7^ Department of Orthopaedic Surgery Aspetar Hospital Doha Qatar

**Keywords:** ACL, failure, knee, risk factor, smoke, tobacco

## Abstract

**Purpose:**

To determine whether patients actively smoking at the time of primary anterior cruciate ligament reconstruction (ACLR) have higher failure rates, defined as the need for revision ACLR procedures over time, compared to non‐smokers.

**Methods:**

This was a retrospective evaluation on a single‐centre cohort of patients who underwent primary ACLR between 2010 and 2015. Patients were included if they had a minimum follow‐up of two years and were classified according to their smoking status at the time of the surgery. The primary outcome was graft failure, defined as the need for revision ACLR surgery due to symptomatic ACL instability. Additionally, subgroup analyses were performed on patient groups known to be at high risk of ACLR failure.

**Results:**

The cohort consisted of 537 patients, including 96 (18%) smokers and 441 (82%) non‐smokers, with a mean follow‐up of 56 months (range 24–89). Demographic characteristics were similar between smokers and non‐smokers at baseline. Overall, 22 patients (4.1%) underwent revision ACLR. The failure rate was significantly higher in smokers (9.4%, 9/96) compared to non‐smokers (2.9%, 13/441) (*p* = 0.008, odds‐ratio (OR): 3.41, 95% confidence interval (CI): 1.41–8.22). This difference was even greater in specific subgroups: smokers had significantly higher failure rates than non‐smokers among patients under 25 years of age (31% vs. 9.8%, *p* = 0.006, OR: 4.13, 95% CI: 1.54–11.07), highly active patients (20.7% vs. 7.7%, *p* = 0.044; OR; 3.13, 95% CI: 1.01–9.30) and male patients (13.3% vs. 3.9%, *p* = 0.010; OR: 3.83, 95% CI: 1.41–10.40).

**Conclusions:**

Smoking at the time of ACLR was associated with a threefold increased risk of ACLR failure. The ACLR failure risk was even greater in patients under 25 years of age, male and those participating in high‐activity sports. Notably, a failure rate of 31% was observed in smokers under 25 years of age.

**Level of Evidence:**

Level IV, case series with no comparison group.

**Abbreviations**
ACL: anterior cruciate ligament
ACLR: anterior cruciate ligament reconstruction
ALL: antero‐lateral ligament
BMI: body‐mass index
CI: confidence interval
DVT: deep vein thrombosis
LEAP: lateral extra‐articular procedures
LET: lateral extra‐articular tenodesis
MCL: medial collateral ligament
MRI: magnetic resonance imaging
NS: non‐smokers
OR: odds ratio
S: smokers
SD: Standard Deviation
SNQ: signal‐to‐noise quotient
SSD: side‐to‐side difference
VTE: venous thromboembolism

## INTRODUCTION

Anterior cruciate ligament (ACL) tears are considered one of the most common orthopaedic injuries with an incidence of 68.6 per 100,000 persons every year in the general population, reaching values of 241.0 per 100,000 in males between 19 and 25 years of age [40]. Nonetheless, despite significant improvements both in the understanding of this pathology and in the surgical techniques, ACL reconstruction (ACLR) failure is still a major concern.

Research has recently focused on the identification of factors increasing the risk of ACLR failure. These can result from technical errors during the primary ACLR, environmental factors (e.g., return to pivoting sports) or patients' predisposing factors, which can be either non‐modifiable (e.g. age, sex and specific anatomies) or modifiable (mainly body mass index [BMI] and smoke habit) [8, 9, 29]. Indeed, smoking is widely recognised as a risk factor for serious general health issues but several detrimental effects on the musculoskeletal system have also been reported [2]. Specifically, tobacco smoking has been linked to higher risks of general musculoskeletal complications [46], fracture non‐union and postoperative infections [42], greater knee cartilage loss [3] and worse clinical outcomes after shoulder [41], hip [18, 22], knee [4, 18, 21, 31, 43] and ankle [13, 20] procedures.

As for the ACL, consistent evidence has described worse subjective clinical outcomes after ACLR procedures in smokers [1, 10, 14, 25, 26, 27, 30, 32, 36, 37, 44] while objective evidence is more limited. Three studies have reported greater side‐to‐side difference (SSD) in anteroposterior knee laxity in smokers after ACLR [25, 26, 27] while three different studies have found higher rates of infections, deep vein thrombosis (DVT) and venous thromboembolism (VTE) in smokers [5, 6, 11]. Furthermore, to date, only three studies have described the different failure rates in smokers and non‐smokers that underwent ACLR, all reporting higher failure rates in smokers, thus proposing tobacco consumption as a relevant risk factor for ACLR failure [6, 7, 34]. Nonetheless, two [6, 34] of those three studies did not report specific failure rates in smokers and non‐smokers and collected data from large databases that included different surgical techniques, while the third study [7] reported on a small cohort of 233 patients treated either with isolated ACLR or with ACLR combined with anterolateral ligament reconstruction, without distinguishing between the two groups.

For this reason, a retrospective analysis of a large, homogeneous, single‐centre cohort of patients who underwent primary ACLR with a consistent surgical technique was performed, comparing the failure rates between smokers and non‐smokers at a minimum of two years of follow‐up. The hypothesis was that smokers would have higher failure rates after ACLR, possibly due to the negative effect of smoking on ligament healing.

## METHODS

### Patients' selection and study design

This study is a single‐centre, two‐surgeon, retrospective analysis of prospectively collected data. The study cohort consisted of patients with ACL injury who received a primary ACLR surgery between 2010 and 2015. The clinical research ethics committee of Antwerp University Hospital (UZA) approved the study (B300201628152). All patients provided informed consent to participate in the study, as well as for the evaluation and publication of their results.

Included patients underwent surgery by two senior surgeons (KCL and PV) with isolate ACLR with autologous quadrupled hamstring tendons grafts, without the addition of any lateral extra‐articular procedure (LEAP).

Patients of all ages were included in this evaluation if they had completed at least two years of follow‐up. Patients with incomplete reports were excluded. Other exclusion criteria were revision ACLR procedures, multiligament injuries requiring multiligament reconstruction procedures, ACLR with grafts other than autologous hamstring tendons, knee dislocations, chondral and osteochondral lesions requiring specific reconstructive cartilage procedures, major concomitant procedures such as high tibial osteotomy or other knee ligament reconstructions (including LEAP) and patients requiring ACL reconstruction techniques other than the anatomical reconstruction technique described below (i.e., physeal sparing technique). At that time of the surgeries, indications for LEAP were limited to professional athletes competing in pivoting sports, patients with high grade pivot shift, revision ACLR and those with severe concomitant tears such as multiligament knee injuries. Over the course of this study, indications were broadened when LEAP protective effect got more established. In order to obtain a more homogeneous cohort, patients treated with LEAP were excluded from this study. Concomitant meniscal tears and their specific treatment were noted and are reported in Tables 1 and 2.

**Table 1:** Baseline characteristics in the cumulative cohort.

	*N*	Percentage of total
Number of patients	537	
Non‐smokers	441	82%
Smokers	96	18%
Sex		
Male	293	55%
Female	244	45%
Age at surgery (years)		
Mean	33	
<25	151/537	28%
>25	386/537	72%
Months of follow‐up Mean (range)	56 (24‐89)	
Pre‐op Tegner score		
Mean	6,36	
Tegner ≥ 8	172	32%
Tegner < 8	365	68%
Meniscal injury	219	40%
Medial meniscus only	124	23%
Lateral meniscus only	71	13%
Combined medial + lateral meniscus	24	4%
Treatment		
Meniscectomy	179	33%
Suture	40	7%
Reoperations:	77	14%
ACLR revision	22	4%
Re‐op other than revision	55	10%
‐ Cyclops Resection	41	8%
‐ Meniscus Repair	2	0%
‐ Meniscectomy	4	1%
‐ Irrigation and debridement	5	1%
‐ Hardware Removal	1	0%
‐ Osteochondral lesion	1	0%
‐ Synovectomy	1	0%

Abbreviations: ACLR, anterior cruciate ligament reconstruction; *N*, total number; pre‐op, pre‐operative; Re‐op, re‐operation.

**Table 2:** Baseline characteristics comparing smokers and non‐smokers.

	All	Non‐Smokers		Smokers		*p*‐value
All	537	441	100%	96	100%	
Sex						0.085
Male	293	233	53%	60	62%	
Female	244	208	47%	36	38%	
Age at surgery (years)						
Mean (range)	33 (13–61)	33 (13–61)	28%	31 (15–56)	30%	0.045
<25	151	122	72%	29	70%	0.615
>25	386	319		67		
Months of follow‐up: Mean (range)	56 (24–89)	55 (24–89)		58 (24–89)		0.630
Pre‐op Tegner score						
Mean	6.36	6.35	32%	6.37	30%	0.475
Tegner ≥ 8	172	143	68%	29	70%	0.673
Tegner < 8	365	298		67		
Meniscal injury	219 (41%)	178	40%	41	43%	0.672
Medial only	124 (23%)	100	23%	24	25%	
Lateral only	71 (13%)	59	13%	12	12%	
Medial + Lateral	24 (4%)	19	4%	5	5%	
Treatment						
Meniscectomy	179 (33%)	144	33%	35	37%	
Suture	40 (7%)	34	8%	6	6%	
ACLR revision	22 (4%)	13	23%	9	9%	0.008
Re‐op other than revision	55 (10%)	45	10%	10	10%	0.950
Cyclops resection	41 (8%)	34	8%	7	7%	

Abbreviations: ACLR, anterior cruciate ligament reconstruction; Pre‐op, pre‐operative; Re‐op, re‐operation.

The following demographics were recorded: duration of follow‐up, sex (male/female), age at the time of surgery, pre‐injury Tegner Score [28, 45], meniscus status, meniscal treatment and prior contralateral ACL injury.

Patients were then divided into two groups based on their smoking habits at the time of surgery: active smokers, defined as those reporting active consumption of nicotine‐containing smoke, such as from cigarettes, were dichotomously distinguished from non‐active smokers.

The primary outcome parameter was ACLR graft failure, defined as recurrent symptomatic instability associated with magnetic resonance imaging (MRI) evidence of graft rupture thus requiring revision ACLR surgery. In the present cohort, all patients who presented a clinical and radiological evidence of graft re‐tear underwent revision ACLR; therefore, the clinical failure rate matched the revision rate that was therefore considered for analysis. The secondary outcome parameter was the reintervention rate, encompassing all subsequent procedures other than revision ACLR: removal of cyclops/arthrofibrosis, irrigation and debridement, hardware removal, meniscus reinjury/revision suture/meniscectomy and wound revision.

Subgroup analyses were conducted to evaluate outcomes in specific populations known to be at high risk of ACLR failure: patients under 25 years of age, patients with a high activity level (performing competitive sports with a Tegner score of 8 or higher [45]) before the initial ACL tear and males/females.

### Surgical technique and patients' management

All ACLR procedures were performed by two senior surgeons (PV and KCL) with a consistent technique of anatomical ACLR that has been described previously [33]. Briefly, the ACL graft consisted of a quadrupled semitendinosus tendon (generally resulting in an 8 to 10 mm thickness), positioned in the native ACL anatomical tibial and femoral insertions, fixed in an independently drilled femoral tunnel with a suspension system (Endobutton, Smith & Nephew, USA) and in the tibial tunnel, with an interference screw (BioRCI, Smith & Nephew, USA) and a fixation post. Meniscal lesions were treated accordingly with meniscectomy or suture. All patients were prescribed with the same postoperative protocol and standard ACLR rehabilitation protocol, consisting of partial weight‐bearing with two crutches for 2 weeks, a knee brace used until 4 weeks postoperatively, with no restriction of range of motion. When meniscal repair was performed, a knee brace with restricted 0°–90° range of motion was prescribed for 6 weeks together with partial weight bearing with crutches for 4 weeks post op. Activities such as swimming and cycling were permitted at 6 weeks, running was possible after 3 months, while a gradual return to sport was generally suggested at 6 months after the procedure.

All selected patients who underwent primary ACLR were preoperatively and postoperatively evaluated at standard intervals. For longer‐term follow‐up (>24 months), patients were contacted by phone, email and text message multiple times. Patients were specifically asked if they had undergone revision ACLR in any institution after the initial ACLR procedure. The response rate was 95.8% (537/560). The remaining 4.2% (23 patients) could not be contacted and were considered lost to follow‐up.

### Outcome analysis

Statistical analysis was performed using SPSS (Version 29). Patient demographics were compared between Group S (smokers) and Group NS (non‐smokers) using the independent samples t‐test for continuous variables (age, activity score and follow‐up time) and the chi‐square test for categorical variables such as gender, meniscal injury, prior ACL injury and reoperation. The Fisher's exact test was used to assess the statistical significance of ACLR failure rates between the two groups, as the assumptions of the Chi‐Square test were violated due to low expected counts in some cells. Additionally, the odds ratio (OR) and 95% confidence interval (CI) were calculated to quantify the association between smoking status and ACLR failure. These calculations were repeated in the subgroup analyses. A *p*‐value < 0.05 was considered statistically significant.

A post hoc power analysis was additionally performed to check whether the number of available patients was sufficient to detect a significant difference in ACLR failure rates between smokers and non‐smokers. The analysis was performed using two different statistical analysis: Cohen's h effect size, using a chi‐squared goodness‐of‐fit test at a significance level of *α* = 0.05, and the two‐proportion Z‐test. The post hoc power analysis assessed whether the included patients were sufficient to detect a significant difference in ACLR failure rates between smokers and non‐smokers. Considering the number of patients and failure rates reported in the manuscript, the Cohen's *h* effect size analysis calculated a statistical power of 99.92%, while the two‐proportion Z‐test yielded a power of 75.6%.

## RESULTS

### Demographics

A total of 770 ACLR procedures were performed during the study period. After excluding non‐primary procedures, multiligament injuries, and cases with concomitant procedures that represented exclusion criteria, 748 patients remained for inclusion. An additional 211 patients were excluded since they were treated with ACLR in association with LEAP. As a result, 537 patients were included in the study, with 96 (18%) assigned to Group S (active smokers at the time of primary surgery) and 441 (82%) to group NS (non‐smokers). Mean follow‐up was 56 (24–89) months and did not differ between Group S (58 months, 24–89) and NS (55 months, 24–89) (*p* = 0.63) (Table 1). There was a slightly higher proportion of male patients in the Group S (62.5% compared to 53% in NS), but it was not statistically significant (*p* = 0.085). Mean age at time of surgery was of 31 (range 15–56, SD 10.1) years in Group S and 33 (range 13–61, SD 11.0) years in NS (*p* = 0.045). All other demographic characteristics (sex, mean follow up, Tegner score and associated meniscal injuries) were evenly distributed with no statistically significant difference between the two groups, as seen in Table 2.

In the overall cohort, ACLR failure rate was observed in 4.1% (22/537) of treated patients. However, this rate was significantly higher in Group S (9.4%, 9/96), compared to Group NS (2.9%, 13/441) (*p* = 0.008) (Table 3). Similarly, in high activity patients, defined as those with a pre‐injury Tegner score of 8 or higher, the failure rate was 20.7% in Group S (6/29) versus 7.7% in group NS (11/143) (*p*‐value = 0.044), depicting a statistically significant impact of smoking. When considering patients under 25 years of age at the time of surgery, smokers were found to have a significantly higher failure rate (31%, 9/29) compared to non‐smokers (9.8%, 12/122) (*p* = 0.006). Male patients also demonstrated higher revision rates in smokers (8/60, 13.3%) compared to non‐smokers (9/233, 3.9%), (*p* = 0.010). The analysis showed no significant differences in failure rates based on smoking habits in female patients: 2.8% (1/36) in smokers compared to 1.9% (4/208) in non‐smokers (*p* = 0.553).

**Table 3:** Failure rates intended as subsequent revision ACLR rates.

	Smokers + Non‐smokers	Smokers	Non‐smokers	*p*‐value	Odds ratio
Cumulative cohort	22/537 (4.1%)	9/96 (9.4%)	13/441 (2.9%)	0.008	3.41 (1.41–8.22)
Age < 25 years	21/151 (13.9%)	9/29 (31.0%)	12/122 (9.8%)	0.006	4.13 (1.54–11.07)
Tegner ≥ 8	17/172 (9.9%)	6/29 (20.7%)	11/143 (7.7%)	0.044	3.13 (1.01–9.30)
Male	17/293 (5.8%)	8/60 (13.3%)	9/233 (3.9%)	0.010	3.83 (1.41–10.40)
Female	5/244 (2%)	1/36 (2.8%)	4/208 (1.9%)	0.553	1.46 (0.16–13.42)

Smoking at the time of the initial surgery was therefore found to be a risk factor for ACLR failure both in the cumulative cohort and in the specific subgroups: smokers had a three times greater risk of failure in the cumulative cohort (OR: 3.41; 95% CI 1.41–8.22) and even greater risks if being under 25 years of age (OR: 4.13; 95% CI 1.54–11.07), and of male sex (OR: 3.83; 95% CI 1.41–10.40). Similarly, smoking habit was found to be a risk factor for failure in highly active patients, considered as those with a Tegner score of 8 or more before the initial ACL tear (OR: 3.13; 95% CI 1.01–9.30).

Reoperations other than ACLR were performed in 55 patients (10.2%) and mainly consisted of cyclops removal (44/537, 7.6%). No significant differences were found between smokers and non‐smokers (10.4% vs. 10.2% respectively, *p* = 0.950) in the frequency of reoperations other than revision surgery.

## DISCUSSION

The main finding of this study was that the failure rate of patients who underwent primary ACLR, defined as the need for revision ACLR, differed according to their smoking status at the time of surgery. In our cohort of 537 patients followed for at least two years after surgery, active S had a statistically significant higher failure rate (9.4%) compared to a 2.9% rate found in NS. This difference was even greater when specifically considering patients under 25 years of age (31% in S compared to 9.8% in NS), highly active patients with a pre‐injury Tegner score of 8 or higher (20.7% in S compared to 7.7% in NS) and male patients (13.3% in S vs. 3.9% in NS). As a result, smoking at the time of the initial ACLR procedure was linked to a threefold higher risk of subsequent failure, defined as the need for revision ACLR, in the cumulative cohort that reached a fourfold increase in smokers under 25 years of age.

Smoking is generally considered the leading cause of preventable diseases worldwide, since almost one out of three men regularly smokes, and seven million deaths every year are associated with tobacco consumption [12, 39]. Active smoking habit has also been reported to be a major risk factor in the orthopaedic practice given that a higher risk of surgical complications, faster disease progression and lower clinical outcomes after several surgical procedures have been found in smokers [3, 18, 42, 46]. As for the knee joint, Kanneganti et al. performed a systematic review on the effects of smoking on ligaments and reported that seven out of the eight studies on the topic found a negative association with smoking, either molecularly, biomechanically, or clinically [24]. Specifically, two preclinical studies, published from the same group, evaluated the effect of smoking on medial collateral ligament (MCL) healing in a mouse model: mice exposed to smoking presented delayed and deficient early healing (demonstrated by decreased cellular density and type I collagen expression at seven days after performing a MCL tear) [17] and their ligaments, appeared weaker and less stiff when compared to mice not exposed to smoking [49]. Clinically, smoking has been seen to have a significant detrimental role in ACL pathology: smokers, after ACLR surgery, present worse clinical outcomes [1, 10, 25, 26, 27, 30, 32, 36, 37], lower rates of return to pre‐injury activity [10, 14, 25, 36, 44], higher side‐to‐side differences in anteroposterior knee laxity [25, 26, 27] and higher incidence of infections and DVTs and VTEs [5, 6, 11]. To date, only three studies have reported the impact of smoking on the failure rates of primary ACLR procedures: [6, 7, 34] Cancienne et al. were the first to report on the topic, performing a matched cohort analysis using a national insurance database of patients who underwent ACLR: the authors matched 1659 patients with documented tobacco use to 11,699 controls and found that smokers had a significantly higher incidence of infections (2.0% vs. 0.9% respectively; odds ratio [OR]: 2.3), and a significantly higher rate of venous thromboembolism (1.0% vs. 0.5% respectively; OR: 1.9) [6]. Furthermore, the authors found a significantly higher rate of subsequent ACLR (either ipsilateral or contralateral within the postoperative 4‐year time limitation of the database) in tobacco users compared with matched controls (12.6% vs. 7.8%, respectively; OR: 1.7) [6]. However, it is worth noting that a specific analysis distinguishing ipsilateral and contralateral subsequent ACLR procedures was not reported in their study. In contrast, Lu et al. performed a similar database analysis on 1497 patients with a diagnosis of ACL rupture between 1990 and 2016 and a minimum 2‐year follow‐up after reconstructive procedure to identify common risk factors for both graft failure and contralateral ACL injury, distinguishing between the two conditions [34]. The group found that, among several well‐known factors such as younger age at surgery, lower BMI, earlier return to sport, and allograft use, active smoking history was a predictor for both ipsilateral failure and contralateral rupture, although they did not report specific failure rates nor odds ratios.

Furthermore, the cohorts described in the two studies from Cancienne et al. and Lu et al. [6, 34] were not single‐centre, homogeneous cohorts like the one reported in this manuscript, but rather cohorts coming from large national databases mixing possible confounding factors such as different surgical techniques and different post‐operative protocols. These specific limitations were avoided in the study by Chan and Yau that reported the relationship between tobacco use and graft rupture after ACLR in a single cohort of 233 patients who received ACLR with hamstring tendon autografts and were followed for a mean of 20.2 ± 1.9 months postoperatively [7]. The authors found a significantly higher rupture rate in smokers than in non‐smokers (12.8% vs. 4.6%, respectively) and, interestingly, a significantly higher whole‐graft signal‐to‐noise quotient (SNQ) on post‐operative MRIs of smokers compared with nonsmokers (4.7 ± 4.4 vs. 3.3 ± 3.7, respectively) suggesting a less satisfactory ligamentization process in smokers [7]. Nonetheless, the studied cohort was limited in number (233 patients compared with the 537 reported in the present manuscript) and included both patients who underwent isolated ACLR and those treated with a combined ACL and anterolateral ligament (ALL) reconstruction procedures possibly introducing a relevant bias.

Considering these available reports, the strength of this study lies in being the largest comparative analysis to date of patients treated with a single and consistent ACLR technique, not associated with any lateral‐extraarticular procedure (LEAP), evaluating the different failure rates between smokers and non‐smokers. Beyond reporting an overall three‐fold higher failure rate in smokers, a separate analysis on high‐risk patients was also performed. Indeed, extensive research found higher failure rates in young [15, 16, 23, 48] and highly active patients [23] while the impact of sex is still debated [35, 38]. This was confirmed in our study, given that the failure rates in patients over 25 years of age and those preforming non‐competitive sports, were found to be lower than those found in patients younger than 25 years of age and those performing competitive activities.Considering smoking habits, the differences in failure rates between S and NS were even greater in patients younger than 25 years at the time of surgery, and similar results were found in highly‐active patients. Regarding sex, smoking was found to be associated with significantly higher failure rates in male patients (13.3% vs. 3.9%), but not in female patients (2.8% vs. 1.9%). This could be linked to the overall low number of female smokers (6.7% of the whole cohort) and the very limited number of failures in female patients (2%). On the other hand, no significant differences were found in the rate of reoperations other than revision reconstruction, mainly represented by cyclops removals, between the two groups (10.4% in S vs. 10.2% in NS).

As for age, cumulative failure rate in patients under 25 years was 13.9% (21/151 patients), which is in line with the 11.4% (34/298 patients) rate found in the patients aged 14–25 years treated with ACLR without lateral extra‐articular tenodesis (LET) in the STABILITY randomised clinical trial [16]. It is worth noting that even young patients undergoing ACLR without LET could decrease their failure rate to 9.8% if smoking habit was not present and that, on the contrary, the combination of being younger than 25 years and being an active smoker led to a dramatic 31% failure rate after ACLR without LET.

Nonetheless, this study has several limitations. First, it is a retrospective analysis of prospectively collected data on a cohort of patients who underwent ACLR in a single centre and were exclusively distinguished between active smokers and non‐smokers at the time of surgery, without collecting any quantitative data on the number of cigarettes smoked per day nor on prior smoking habits. It is worth mentioning that a limited percentage of patients (24/96, 25%) in the active smokers group reported having stopped smoking after the ACLR procedure and that none of these patients suffered ACLR failure at the present follow‐up. Despite being preliminary evidence on a limited number of patients, this finding could suggest that smoking cessation may help reduce ACLR failure in patients who smoke at the time of surgery. A second limitation of this study lies in the fact that the prevalence of several other risk factors for ACLR failure such as baseline high‐grade instability, increased tibial slope, pre‐operative hyperlaxity, and time to return‐to‐sport was not reported between the two groups. This could partially limit the quality of the presented results. Third, the present cohort was limited to 537 patients since we decided to exclusively include patients who did not receive any lateral extra‐articular procedure (LEAP), which, in our centre, now represents the majority of patients undergoing ACLR procedures. This was done because, at the time of surgery for this present cohort (2010–2015), only a limited number of patients received LEAP, given that the evidence on the efficacy of such procedure and its optimal indication was just emerging. Furthermore, the failure rates of the patients receiving ACLR + LEAP in our centre are now significantly lower than those reported in this study especially when considering the 13.9% and 9.9% cumulative failure rates found in patients younger than 25 years old and in highly active patients, respectively. For this reason, a currently unmet large number of patients would now be needed to detect any significant difference between smokers and non‐smokers treated with ACLR + LEAP. Additionally, an important limitation that must be noted is that the follow‐up evaluations were limited to clinical informations contained in questionnaires collected via phone calls and emails, while radiological and physical examinations are lacking. As a result, failure was defined exclusively as having undergone revision ACLR surgery and not in clinical or radiological confirmation of the graft tear. Lastly, in this cohort, ‘smoking’ was defined and limited to traditional tobacco cigarette consumption, since the now widespread smokeless tobacco and vaping products were commercialised only in the last 10 years. Recent evidence has found contrasting results when comparing smokeless tobacco and conventional cigarette smoke: Holle et al. reported that smokeless tobacco use could lead to even higher risks of complications after ACLR procedures [19] whereas Waters et al. found lower complication rates after total knee replacement surgeries in smokeless tobacco users compared to cigarette smokers [47].

Future studies will therefore have to primarily focus on the protective effect of additional LEAP in smokers to clarify if smoking is a risk factor for ACLR failure also in that specific population. Additionally, prospective analyses should be performed quantifying the number of cigarettes smoked per day to determine if there is any dose‐effect relationship but also discriminating between the different products available on the market, such as traditional cigarettes, smokeless tobacco options, and vaping devices.

## CONCLUSION

In the present study, smoking at the time of primary ACLR surgery was associated with a threefold higher risk of ACLR failure. The ACLR failure risk was even greater in smokers under 25 years of age, in males and in highly active patients. These results may help surgeons better counsel their patients regarding the impact of smoking on the outcome of their ACLR procedure.

## AUTHOR CONTRIBUTIONS

All authors contributed to the study conception and design. Material preparation, data collection and analysis of the available literature were performed by Filip Hendrikx, Pietro Conte, Annemieke Van Haver and Kristien Vuylsteke with the constant supervision of Koen Carl Lagae and Peter Verdonk. The first draft of the manuscript was written by Pietro Conte and Filip Hendrikx. Then, Koen Carl Lagae and Peter Verdonk commented on previous versions of the manuscript and helped refine the manuscript. Finally, all authors read and approved the final manuscript.

## CONFLICT OF INTEREST STATEMENT

The authors declare no conflicts of interest.

## ETHICS STATEMENT

The clinical research ethics committee of Antwerp University Hospital (UZA) approved the study (B300201628152). All the patients signed informed consent to participate in the study as well as for the evaluation and publication of their results.

## Data Availability

Clinical data regarding this study could be shared upon request.
